# Enhancing the activity of β-lactamase inhibitory protein-II with cell-penetrating peptide against KPC-2-carrying *Klebsiella pneumoniae*

**DOI:** 10.1371/journal.pone.0296727

**Published:** 2024-01-26

**Authors:** Chawalit Chatupheeraphat, Jiratchaya Peamchai, Noramon Kaewsai, Nuttapat Anuwongcharoen, Warawan Eiamphungporn

**Affiliations:** 1 Center for Research Innovation and Biomedical Informatics, Faculty of Medical Technology, Mahidol University, Salaya, Nakhon Pathom, Thailand; 2 Department of Clinical Microbiology and Applied Technology, Faculty of Medical Technology, Mahidol University, Bangkok, Thailand; 3 Department of Community Medical Technology, Faculty of Medical Technology, Mahidol University, Salaya, Nakhon Pathom, Thailand; UAE University: United Arab Emirates University, UNITED ARAB EMIRATES

## Abstract

Carbapenem-resistant Enterobacterales (CRE) is considered a paramount threat due to its rapid spread and high mortality rate. *Klebsiella pneumoniae* carbapenemases (KPCs), specifically KPC-2, are prevalent enzymes responsible for carbapenem resistance in many countries. While combinations of antibiotics are commonly used, they must be tailored to match the remaining susceptibility of the infecting strains. Therefore, there is a need to develop the β-lactamase inhibitor to effectively address this issue. β-lactamase inhibitor protein (BLIP) and its variants, BLIP-I and BLIP-II, have demonstrated the ability to inhibit class A β-lactamases. In particular, BLIP-II shows strong binding to the KPC-2 carbapenemase, making it a potential candidate for inhibition. To improve the intracellular penetration of BLIP-II, a cell-penetrating peptide (CPP) was employed. In this study, a KRK-rich peptide was introduced at either the N-terminal or C-terminal region of tBLIP-II, excluding the signal sequence of the BLIP-II protein. tBLIP-II, tBLIP-II-CPP, and CPP-BLIP-II were successfully expressed, and the chimeric proteins retained inhibitory activity compared to tBLIP-II alone. It is apparent that homology modeling demonstrated neither the poly-histidine tag nor the CPP interfered with the essential interaction residues of tBLIP-II. Interestingly, BLIP-II-CPP exhibited the highest inhibitory activity, reducing the minimal inhibitory concentration (MIC) of meropenem by 8 folds. Moreover, the combination of tBLIP-CPP with meropenem significantly decreased the viable bacterial cell count compared to the combination of tBLIP-II with meropenem or meropenem alone. These findings suggest that tBLIP-CPP is a promising candidate for restoring carbapenem susceptibility against CRE and provides a valuable therapeutic option for infections caused by CRE.

## Introduction

Antimicrobial resistance is a critical global health issue contributing to mortality. The estimation reveals that antimicrobial resistance organisms account for approximately 700,000 deaths annually worldwide, with a projected increase to 10 million deaths per year by 2050 [1, 2]. Furthermore, the emergence of multidrug-resistant (MDR) or extensively drug-resistant (XDR) bacteria, coupled with the diminishing rate of new antibiotic development, poses a severe threat to public health. Bacterial resistance to antibiotics can occur through various mechanisms, including limiting the uptake of a drug, active efflux of a drug, modifying a drug target, enzymatically inactivating a drug, and bypassing a metabolic pathway [3, 4]. Notably, the presence of the CRISPR-Cas system has been found to be highly negatively associated with antibiotic resistance in some bacteria [5, 6]. In particular, The World Health Organization (WHO) has recently listed carbapenem-resistant Enterobacterales (CRE) as a paramount antimicrobial-resistant menace due to its rapid dissemination, propensity for multidrug resistance, and elevated mortality rate [7, 8]. The production of carbapenemases that hydrolyze the β-lactam ring of carbapenems is the major mechanism underlying carbapenem resistance in Enterobacterales. Carbapenemases belong to molecular classes A, B, and D β-lactamases. Class A and D enzymes operate through a serine-based hydrolytic mechanism, whereas class B enzymes are metallo-β-lactamases that feature zinc in their active sites [9, 10]. Among the carbapenemases, *Klebsiella pneumoniae* carbapenemases (KPCs) are currently the most clinically significant enzymes among the class A carbapenemases worldwide [11]. Their discovery dates back to 1996, and subsequently, they have spread globally across continents [12]. Among the KPCs, KPC-2 is the most prevalent enzyme, demonstrating a wide-ranging substrate profile encompassing penicillin, cephalosporins, and carbapenems. Moreover, KPC-2 can confer resistance to common β-lactamase inhibitors, such as clavulanate, sulbactam, and tazobactam [13–16]. Recently, a β-lactamase inhibitor named avibactam has been introduced, exhibiting the capability to deactivate the KPC-2 enzyme [17–19]. Notably, the combination of ceftazidime with avibactam renders most CRE susceptible to treatment, offering a potential alternative regimen for CRE infections. Nevertheless, reports have emerged regarding mutations in the KPC-2 enzyme, resulting in resistance to ceftazidime-avibactam [13, 20–22]. Therefore, the pressing need remains for the development of a novel β-lactamase inhibitor to effectively address this concern.

The β-lactamase inhibitor protein (BLIP) was originally isolated from the bacterium *Streptomyces clavuligerus*, renowned for its clavulanic acid production, in 1990. BLIP possesses the capacity to bind and inhibit β-lactamases including TEM-1, widely found in Gram-negative bacteria and KPC-2, a β-lactamase capable of hydrolyzing nearly all clinically relevant β-lactam antibiotics [23–25]. In addition to BLIP, *Streptomyces exfoliates* has yielded two more β-lactamase inhibitor proteins, denoted as BLIP-I and BLIP-II. BLIP-I shares a 38% sequence similarity with BLIP and employs a comparable mechanism to exert its inhibitory effects on β-lactamases. Conversely, despite the absence of sequence similarity with BLIP and BLIP-I, BLIP-II exhibits notable binding similarities to TEM-1 [26]. BLIP-II effectively inhibits the TEM-1 β-lactamase with an exceptionally low K value. Moreover, an examination of the interaction rate constants, *k*_on_ and *k*_off_, was undertaken to evaluate the potency of BLIP-II as an inhibitor of the KPC-2 carbapenemase, elucidating a remarkably robust interaction between BLIP-II and KPC-2 [27]. Importantly, BLIP-II demonstrates an approximately 100-fold greater affinity for binding to KPC-2 when compared to BLIP. Despite its highly potent inhibition of KPC-2 β-lactamase, the administration of BLIP-II in combination with a carbapenem antibiotic as a therapeutic agent faces challenges. One of the limitations is a BLIP-II molecule that would not be expected to penetrate the bacterial outer membrane. Additionally, the hydrophilic nature of the protein presents a constraint on its cellular uptake owing to the hydrophobic properties of the cell wall and membrane [16]. To effectively target intracellular sites, the inhibitor must successfully traverse the bacterial cell. Accordingly, the aid of facilitating molecules, such as cell-penetrating peptides (CPPs) could be employed to deal with this obstacle.

Cell-penetrating peptides or CPPs are a class of short-cationic and amphipathic peptides known for their ability to traverse the cell membrane and mediate the transport of specific cargo molecules into cells [28]. In recent years, CPPs have gained considerable attention as a promising approach for drug delivery, offering a means to transport various therapeutic agents including antimicrobial peptides (AMPs), small molecules, nucleotides, and proteins [29, 30]. Traditionally, CPPs are defined as peptides comprising 5–30 amino acids and characterized by a net positive charge, primarily attributed to the presence of lysine (K) and arginine (R) residues [31]. CPPs that are arginine- and lysine-rich cationic peptides can readily enter cells not only by themselves but also by carrying other macromolecular cargos [32, 33]. The KRK motif, which encompasses sequences such as CGKRK, Pep-1, and KRK, is frequently observed in a wide range of CPPs [34–36]. Notably, through the utilization of Random Forest-based prediction models, specific dipeptides (RR, KK, KR, RK) and tripeptides (RRR, KKK, KKR, KRK, RKK) have been identified as significant determinants contributing to the cell-penetrating properties of peptides, underscoring the importance of diverse sequential features [37]. An example of a CPP is pVEC, which comprises a series of positively charged residues (LLIILRRRIRKQAHAHSK) derived from the adhesion molecule vascular endothelial cadherin [23]. Interestingly, a previous study demonstrated that a chimeric protein constructed by the addition of five N-terminal residues (LLIIL) of pVEC to the N-terminus of the Ala46-Tyr51 β-hairpin loop of BLIP exhibited competitive inhibition of TEM-1 β-lactamase with a K value of 58 μM [38]. In this study, we aimed to enhance the permeability of BLIP-II, which showed superior inhibitory activity, by introducing a KRK-rich peptide to either the C-terminus or N-terminus of BLIP-II. Our objective was to facilitate its effective penetration into intracellular targets. These chimeric proteins were expressed *in vitro* and investigated their carbapenemase inhibitory activity in KPC-2-carrying *K*. *pneumoniae*.

## Materials and methods

### Homology modeling

The sequence of the BLIP-II gene from *S*. *exfoliatus* was obtained from NCBI (accession number AAC35427.1). The sequence encoding the signaling peptide at residues 1–40 of BLIP-II was excluded to prevent protein secretion from the host, resulting in a truncated BLIP-II (tBLIP-II) [39]. The DNA sequence encoding the 9 residues cell-penetrating peptide (KRKKRKKRK) was aligned either at the C-terminus or the N-terminus of tBLIP-II, designated as tBLIP-II-CPP or CPP-tBLIP-II, respectively. tBLIP-II and tBLIP-II-CPP were tagged with 6×-His sequence at the N-terminus, while CPP-tBLIP was tagged with 6×-His sequence at the C-terminus. The prediction of 3D models for designed proteins was carried out using the I-TASSER server. The level of confidence in the predicted model was quantitatively assessed based on the C-score [40–42] and only the highest C-score models were selected. Furthermore, The stereochemical quality of the predicted models was evaluated using PROCHECK [43], ProSA [44], and Pro-Q [45]. Finally, the 3D structural visualization was performed using open-source PyMOL [46].

### Construction of chimeric genes encoding tBLIP-II, tBLIP-II-CPP, and CPP-tBLIP-II

The DNA fragments of tBLIP-II, tBLIP-II-CPP, and CPP-tBLIP-II were commercially synthesized and cloned into pETDuet-1 by Genscript (Piscataway, NJ, USA). It should be noted that the expression of BLIP-II may inhibit β-lactamase, which could affect the functionality of ampicillin used as a selection marker. To address this, all constructs were additionally cloned into another vector, pACYCDuet-1, which carries a chloramphenicol-resistant gene as the selection marker. Polymerase chain reaction (PCR) was employed to introduce the *BamH*I and *Hind*III restriction sites for the tBLIP-II and tBLIP-II-CPP constructs, and the *Nco*I and *Hind*III restriction sites for the CPP-tBLIP-II construct, using the primers listed in Table 1. All specific primers were ordered from Integrated DNA Technologies, Inc. (Coralville, IA, USA). The PCR products and pACYCDuet-1 were digested with the corresponding restriction enzymes and then ligated together using T4 DNA ligase (New England Biolabs Inc., Ipswich, MA, USA). The resulting ligation mixtures were transformed into NovaBlue *Escherichia coli* competent cells from Novagen (Darmstadt, Germany) and selected on Luria-Bertani (LB) agar plates supplemented with 34 μg/mL of chloramphenicol (Tokyo Chemical Industry Co., Ltd., Tokyo, Japan). The presence of the inserted gene was verified by DNA sequencing.

**Table 1:** Oligonucleotides used in this study.

Construct	Primer	Sequence**
**tBLIP-II**	*BamH*I tBLIP-II for*	5’- CA**GGATC****C**AGCGACCAGCGTTGTGG-3’
	*Hind*III tBLIP-II rev	5’-GC**AAGCTT**TCAACCTTTCAGCGCATAC-3’
**tBLIP-II-CPP**	*BamH*I tBLIP-II-CPP for*	5’- CA**GGATCC**AGCGACCAGCGTTGTGG-3’
	*Hind*III tBLIP-II-CPP rev	5’-GC**AAGCTT**TCATTTGCGTTTTTTGCGTTTC-3’
**CPP-tBLIP-II**	*Nco*I CPP-tBLIP-II for	5’-TA**CCATGG**GTAAGCGTAAGAAGCGTAAAA-3’
	*Hind*III CPP-tBLIP-II rev	5’-CGC**AAGCTT**TTAGTGGTGGTGATGGTGGT-3’

*Forward primers used for constructing tBLIP-II and tBLIP-II-CPP are in the same sequence.

**The restriction sites in the primers were annotated as bold and underlined.

### Expression and purification of tBLIP-II, tBLIP-II-CPP, and CPP-tBLIP-II

The recombination plasmids were transformed into *E*. *coli* BL21 (DE3) (Novagen Inc., Darmstadt, Germany) using the heat-shock method. The transformants were cultured in modified terrific broth (TB) medium supplemented with 34 μg/mL of chloramphenicol at 37°C, 180 rpm until the OD_600_ nm reached 1.0. Isopropyl-β-D-thiogalactoside or IPTG (Bio Basic Inc., Ontario, Canada) was added to the growth culture at a final concentration of 1 mM to induce overexpression of the recombinant protein. The transformants were further incubated at 16°C, 120 rpm for 16 h. Subsequently, the cells were harvested and suspended in a lysis buffer (50 mM phosphate buffer with 300 mM NaCl, pH 7.4), followed by sonication. After centrifugation to remove cell debris, the supernatant was filtered through a 0.45 μm syringe PES filter (Guangzhou Jet Bio-Filtration Co., Ltd., Guangzhou, China) and purified using a prepacked Ni-NTA Sepharose column (Cytiva Life Sciences, Marlborough, MA, USA) with an ÄKTA pure protein purification system (GE Healthcare, Uppsala, Sweden). The recombinant proteins were eluted with gradient imidazole in the same buffer. The imidazole was subsequently removed, and the proteins were concentrated using Macrosep advance centrifugal devices with a 10K MW pore-size membrane (PALL Corp., Port Washington, NY, USA). The molecular weight of the purified proteins under denaturing conditions was determined by SDS-PAGE analysis. The purity of the recombinant protein was assessed based on band intensity using Image Lab software (Bio-Rad Laboratories Inc., Hercules, CA, USA). The protein concentration of the purified proteins was measured using the protein assay dye reagent concentrate (Bio-Rad Laboratories, Inc., Hercules, CA, USA) according to the manufacturer’s protocol, and the proteins were stored in 10% glycerol in a 50 mM phosphate buffer with 300 mM NaCl, pH 7.4 at -80°C until further use.

### β-lactamase inhibition assay

β-lactamase activity was assessed spectrophotometrically by measuring the hydrolysis of nitrocefin as previously described with little modification [47]. Briefly, the reaction mixture consisted of 83 μg of nitrocefin (EMD Millipore Corp., Burlington, MA, USA), 10% glycerol (Bio Basic Inc., Ontario, Canada), 167 mg of bovine serum albumin (Sigma-Aldrich Corp, Burlington, MA, USA), and 0.02 mg/mL of β-lactamase obtained from *Bacillus cereus* 569H (EMD Millipore Corp., Burlington, MA, USA). The activity of β-lactamase was determined by kinetically measuring the absorbance at 490 nm for 60 sec using UV-Vis spectrophotometer (Shimadzu Corp., Kyoto, Japan). To evaluate the inhibitory effects, inhibitors including tBLIP-II, tBLIP-II-CPP, and CPP-tBLIP-II were pre-incubated with the assay mixture without nitrocefin for 15 min at 30°C before adding nitrocefin as the substrate. The concentrations of the inhibitors ranged from 0.0078 μM to 2 μM. The IC_50_ values were determined through direct competition between nitrocefin and protein inhibitors under the aforementioned conditions. The IC_50_ values were obtained by plotting the percent residual enzyme activity on nitrocefin against the inhibitor concentration. The IC_50_ was defined as the concentration of the inhibitor that inhibited the hydrolytic activity of the enzyme by 50%.

### Minimum inhibitory concentration (MIC) determination

*K*. *pneumoniae* ATCC BAA-1705 carrying *bla*_KPC-2_, and *K*. *pneumoniae* ATCC BAA-2472 carrying *bla*_NDM-1_ were obtained from American Type Culture Collection (ATCC). Both strains harboring carbapenem resistance genes were confirmed using PCR with specific primers as previously described [48]. The MICs of meropenem alone or in combination with tBLIP-II, or tBLIP-II-CPP against *K*. *pneumoniae* ATCC BAA-1705 and *K*. *pneumoniae* ATCC BAA-2472 were determined using the broth microdilution method followed CLSI recommendations [49]. Briefly, bacterial suspensions from freshly grown mid-log phase cultures were diluted to a concentration of 10^6^ CFU/mL in cation-adjusted Mueller-Hinton broth (CAMHB), and added to each well containing various concentrations of meropenem along with the recombinant proteins. The final concentrations of meropenem ranged from 256 to 1 μg/mL, with fixed concentrations of 10 μM and 20 μM for tBLIP-II, or tBLIP-II-CPP. The suspensions were then incubated for 20–24 h at 37°C. The MIC was determined as the lowest concentration of meropenem alone or combined agents at which there was a complete absence of bacterial growth.

### Time-kill kinetics assay

A growth kinetic analysis was carried out following a previously described method with slight modifications [38]. In brief, tubes containing freshly prepared CAMHB, supplemented with 2 μg/mL of meropenem alone or in combination with 10 μM of recombinant proteins, were inoculated with *K*. *pneumoniae* ATCC BAA-1705 to a density of 10^6^ CFU/mL in a final volume of 5 mL. The untreated tube was also conducted as a control. The tubes were then incubated in an incubator shaker at 37°C. At designated time points (0, 1, 2, 3, 4, 6, 8, and 24 h post-inoculation), aliquots were withdrawn and serially diluted in saline to determine viable counts. Diluted samples (50 μL) were plated on Mueller-Hinton agar (MHA) plates, and bacterial counts were determined after 18 h of incubation at 37°C. The results were expressed as log10 of viable cell numbers (CFU/mL) at each time point.

### Cell viability assay

To investigate the toxicity of recombinant proteins, the human lung fibroblast cell line MRC-5 (CCL-171) was treated with recombinant proteins. MRC-5 cell line was obtained from ATCC and cultured in Eagle’s minimum essential medium (EMEM, Gibco Inc., Billings, MT, USA) supplemented with 10% fetal bovine serum (FBS, Gibco Inc., Billings, MT, USA), 100 units of penicillin, and 100 μg/mL of streptomycin (Gibco Inc., Billings, MT, USA). The cells were maintained in a controlled environment at 37°C with 5% CO_2_. To determine cell viability, 10,000 MRC-5 cells were seeded into a 96-well plate and incubated at 37°C with 5% CO_2_ for 24 h. Subsequently, the culture medium was replaced with a fresh medium containing varying concentrations of recombinant proteins (6.25, 12.5, 25, 50, 100 μM) and incubated for an additional 24 h. After the incubation period, 10 μL of a 5 mg/mL MTT solution (3-(4,5-Dimethylthiazol-2-yl)-2,5-Diphenyltetrazolium Bromide) (Invitrogen, Waltham, MA, USA) was added to each well and incubated for 4 h at 37°C. Then, 100 μL of a 10% SDS solution in 0.01 N HCl was introduced to dissolve the formazan crystals. The absorbance was measured at 570 nm using a microplate reader (Tecan Group Ltd., Zurich, Switzerland). The results were expressed as a percentage of cell viability relative to the untreated control.

### Hemolytic activity assay

Sheep erythrocytes were used to evaluate the hemolytic activity of tBLIP-II and tBLIP-II-CPP, as described in a previous study [50]. Briefly, defibrinated sheep red blood cells (sRBCs) were centrifuged at 1,000× g for 10 min at 4°C. The packed sRBCs were washed three times with phosphate-buffered saline (PBS), pH 7.4 and then suspended in PBS to achieve a concentration of 4% (v/v). Serial two-fold dilutions of each protein were prepared, ranging in concentration from 200 to 12.5 μg/mL. Aliquots of the sRBC suspension were mixed with the protein in a 1:1 ratio, resulting in final concentrations ranging from 6.25 to 100 μg/mL. The mixtures were incubated at 37°C for 1 h and then centrifuged at room temperature, 1,000× g for 10 min. The supernatants were transferred to a 96-well microplate, and the absorbance was measured at 540 nm using a microplate reader. sRBCs incubated with PBS and 0.1% Triton X-100 solution were used as blank and positive controls, respectively. Melittin, known for its strong hemolytic activity, served as a positive reference. The percentage of hemolytic activity was calculated using the following equation: Hemolysis (%) = {(Sample absorbance—PBS absorbance)/(0.1% Triton X-100 absorbance—PBS absorbance)} × 100.

### Statistical analysis

Experiments were performed in triplicate at least three different times. The quantitative data are presented as mean ± standard deviation (SD). All results were analyzed for normal distribution using the Shapiro-Wilk test. The null hypothesis was rejected when a probability <0.05. When no significant difference from normality was identified, a comparison of two means was performed by one-way analysis of variance (ANOVA). All statistical calculations were performed using GraphPad Prism version 8.4.3. Values of P <0.05 were considered statistically significant.

## Results

### Homology modeling and quality evaluation of the predicted models

**Fig 1:**
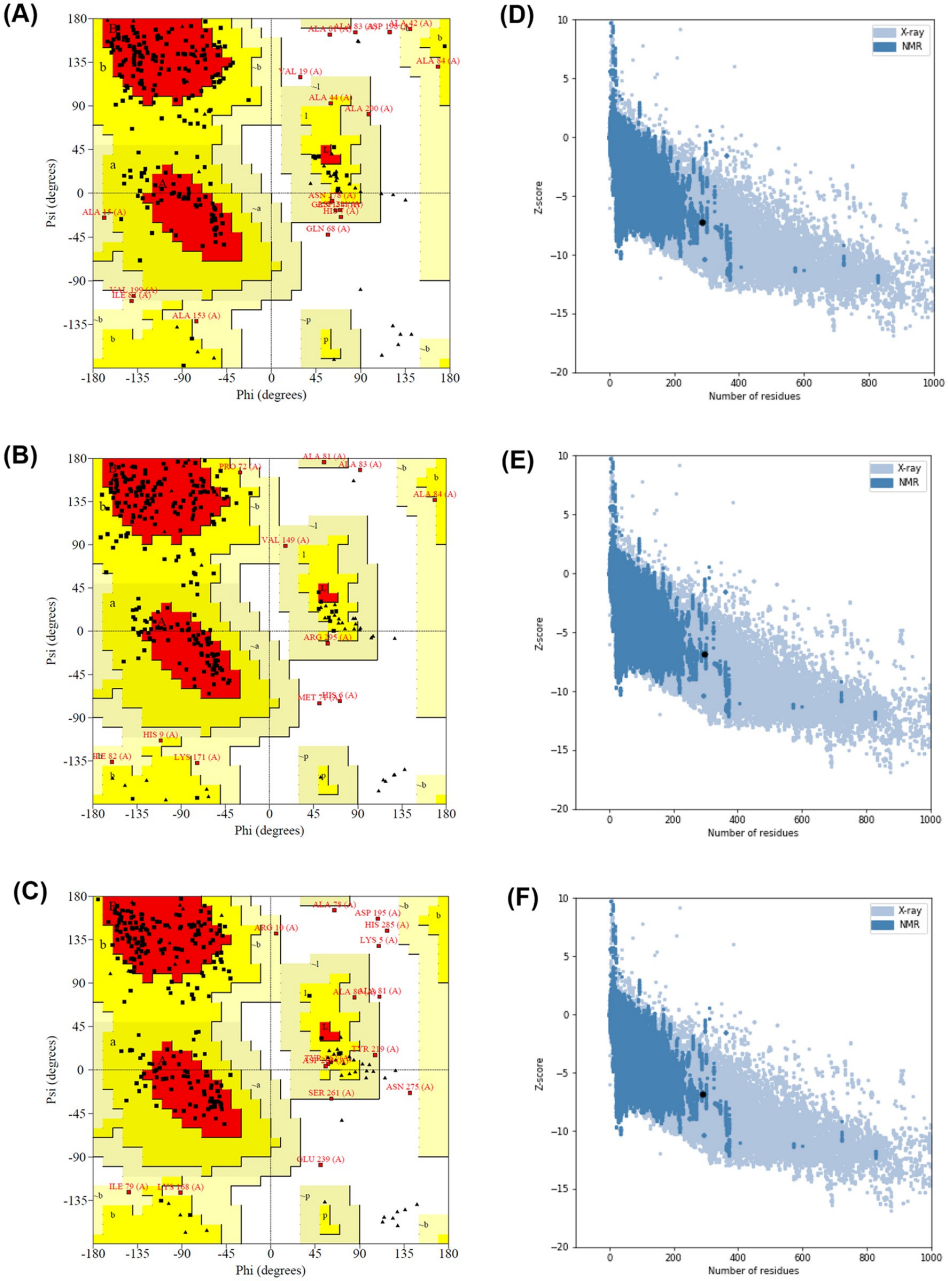
Evaluation of predicted model conformations. (A-C) Ramachandran plots depicting the phi (ϕ) and psi (ψ) dihedral angles of tBLIP-II, tBLIP-II-CPP, and CPP-tBLIP-II, respectively. The Ramachandran plots illustrate residues residing within favored regions (red), allowable regions (yellow), generously allowable regions (light yellow), and disallowed regions (white). (D-F) ProSA Z-score plots of tBLIP-II, tBLIP-II-CPP, and CPP-tBLIP-II, respectively. The ProSA Z-score plots elucidate the distribution of Z-scores (black dots) across a chart encompassing the Z-scores of protein chains obtained from the Protein Data Bank. The blue region signifies Z-scores associated with protein structures characterized by NMR analysis. In contrast, the grey region corresponds to Z-scores of protein structures determined through X-ray diffraction studies.

**Fig 2:**
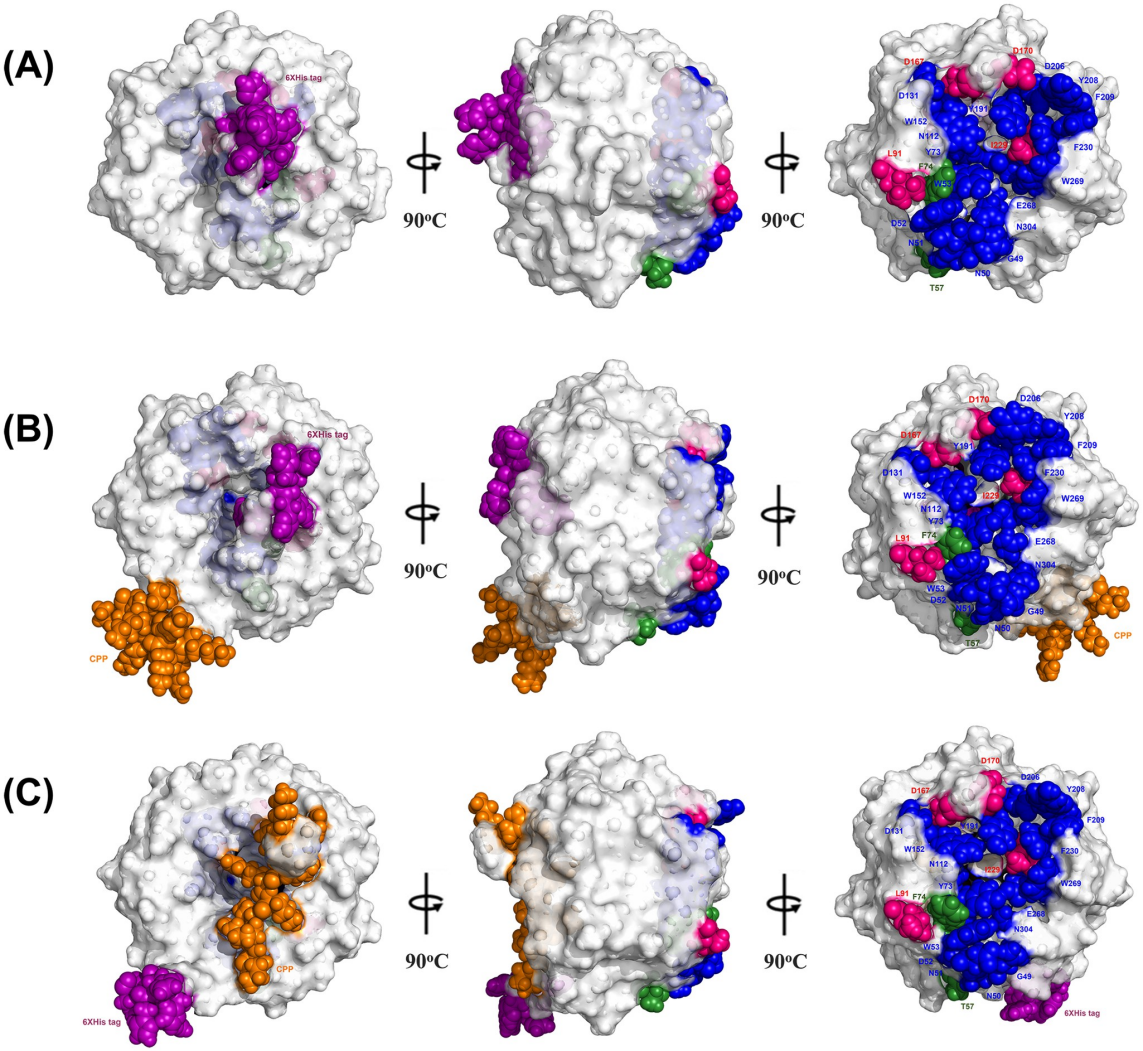
Predicted structures of the recombinant proteins rotating in different angles. (A) tBLIP-II, (B) tBLIP-II-CPP, and (C) CPP-tBLIP-II. Purple and orange spheres represent the His-tag and CPP, respectively. Residues involving specific interaction to TEM-1 and Bla-1 are highlighted with green and pink color, respectively, while blue spheres with corresponding color labels highlight the common interacting residues to both enzymes.

The homology modeling was performed to investigate the effects of CPP and His-tag insertion against the binding interface of the three recombinant proteins. The amino acid sequences of tBLIP-II, CPP-tBLIP-II, and tBLIP-II-CPP were submitted to the I-TASSER web server to generate the homology models. The I-TASSER built-in template search feature identified the top 10 threading crystalized structures of BLIP-II, with 3QHY and 1JTD showing the highest sequence identify of 1.00 and 0.90–1.00 to the threading aligned region, respectively. The C-score was used to determine the confidence of each model. The range was -5 to 2, with higher values indicating greater confidence. The calculated C-scores for tBLIP-II, CPP-tBLIP-II, and tBLIP-II-CPP were 0.94, 0.93, and 0.96, respectively. Structural stability and accuracy were assessed using various tools from Table 2, including PROCHECK to generate a Ramachandran plot for secondary structure analysis. The results showed that over 95% of the amino acid residues were located in allowed regions, with only a small percentage (2.2% in tBLIP-II, 1.2% in CPP-tBLIP-II, and 3% in tBLIP-II-CPP) in disallowed regions (Fig 1A–1C). The Pro-Q tool was utilized to evaluate the models based on residue-specific local quality, as indicated by the LG-score and MaxSub-score. Models with LG-score greater than 3 and a MaxSub-score higher than 0.5 were considered favorable models. The models were evaluated using ProSA, with the crystal structure (3QHY) as the template. The ProSA Z-scores for all three models fell within the range typically observed for native proteins of similar size (Fig 1D–1F). Validation methods confirmed reliable 3D structures for all three proteins. 3D structures were visualized in PyMol, emphasizing the poly-histidine tag (His-tag) represented in purple and the CPP represented in orange (Fig 2 and S1–S3 Files). The significant interaction residues were categorized into three distinct groups: residues interacting with TEM-1, residues interacting with Bla-1, and residues interacting with both TEM-1 and Bla-1, highlighted in green, pink, and blue, respectively. It is noteworthy that neither the His-tag nor the CPP had any impact on the crucial residues responsible for the interaction.

**Table 2:** Assessment of the predicted three-dimensional structures of tBLIP-II, tBLIP-II-CPP, and CPP-BLIP-II.

Validation index	tBLIP-II	tBLIP-II-CPP	CPP-tBLIP-II
**i-Tasser**			
C-score	0.94	0.93	0.96
Estimated TM-score	0.84±0.08	0.84±0.08	0.84±0.08
Estimated RMSD	4.2±2.8 Å	4.3±2.8Å	4.2±2.8 Å
**Ramachandran plot (residues, (%))**			
Residues in most favored regions	150 (64.7%)	167 (69.0%)	169 (71.3%)
Residues in additional allowed regions	65 (28.0%)	65 (26.9%)	53 (22.4%)
Residues in generously allowed regions	12 (5.2%)	7 (2.9%)	8 (3.4%)
Residues in disallowed regions	5 (2.2%)	3 (1.2%)	7 (3.0%)
**ProSA**			
Z-score	-7.22	-6.82	-6.86
**Pro-Q**			
LG-score	7.449	7.678	7.255
MaxSub-score	0.509	0.547	0.508

### Construction, expression, and purification of tBLIP-II, tBLIP-II-CPP, and CPP-tBLIP-II

**Fig 3:**
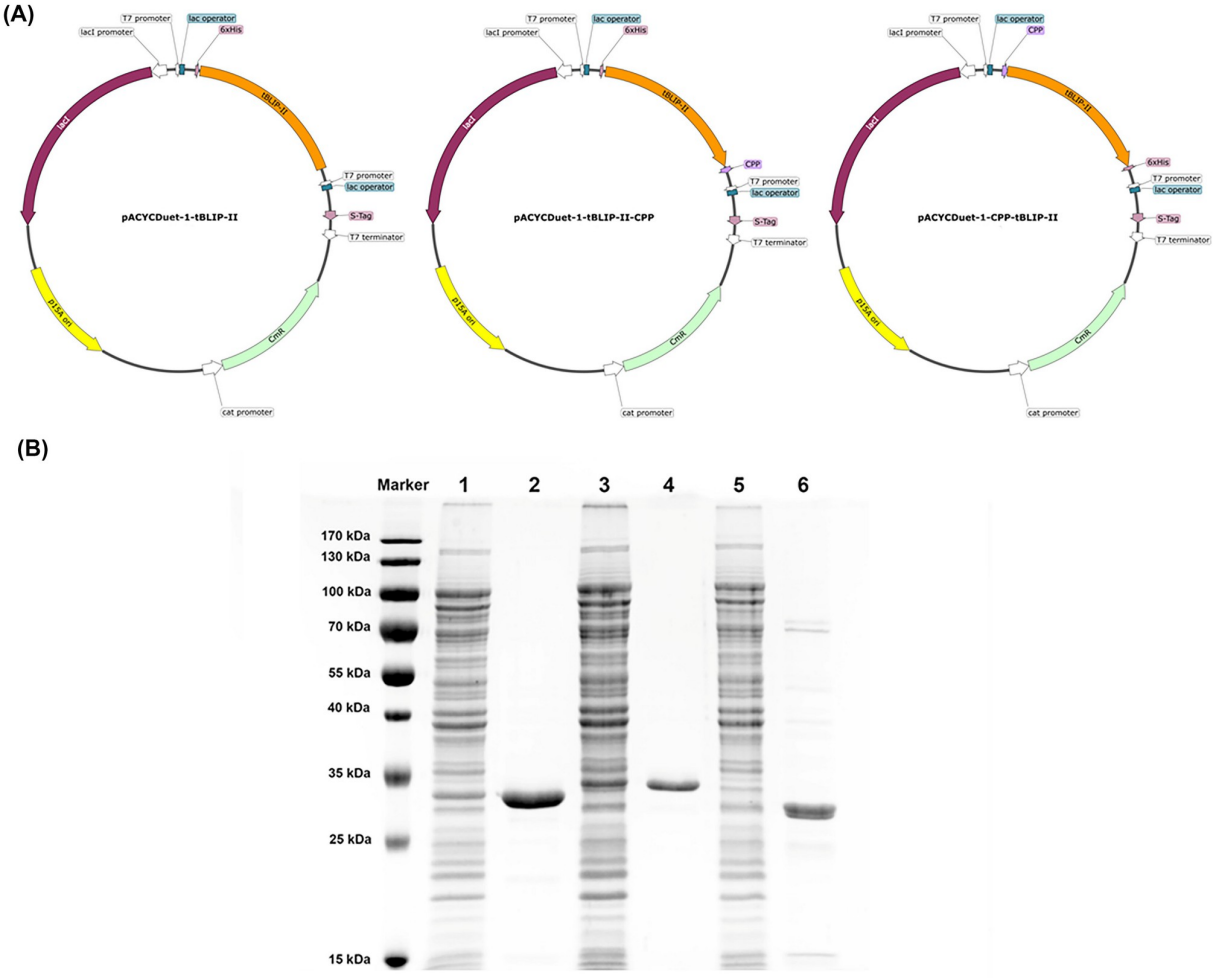
Construction, expression and purification of recombinant proteins. (A) Construction of tBLIP-II, tBLIP-II-CPP, and CPP-tBLIP-II expression vector system based on the pACYCDuet-1. (B) Expression and purification of tBLIP-II, tBLIP-II-CPP, and CPP-tBLIP-II fusion proteins in *E*. *coli* BL21 (DE3). Purified proteins were determined by 10% SDS PAGE analysis Lane are as follows: Lane 1; crude extract of *E*. *coli* expressing tBLIP-II, Lane 2; purified tBLIP-II, Lane 3; crude extract of *E*. *coli* expressing tBLIP-II-CPP, Lane 4; purified tBLIP-II-CPP, Lane 5; crude extract of *E*. *coli* expressing CPP-tBLIP-II, and Lane 6; purified CPP-tBLIP-II.

The pACYCDuet-1-tBLIP-II, pACYCDuet-1-tBLIP-II-CPP, and pACYCDuet-1-CPP-tBLIP-II were successfully constructed, and subsequent protein expression in *E*. *coli* resulted in the production of soluble proteins (Fig 3 and S1 Raw image). The CPP sequence was in-frame fused either at the N-terminus or the C-terminus of tBLIP-II, accompanied by a His-tag at the opposite terminus. Following purification, all proteins exhibited a homogeneity with their respective molecular weights determined under denaturing conditions. The purities of tBLIP-II, tBLIP-II-CPP, and CPP-tBLIP-II were evaluated by assessing SDS-PAGE band intensity, resulting in values of 94.1%, 98.7%, and 83.3%, respectively. Notably, tBLIP-II and tBLIP-II-CPP demonstrated molecular weights approximately of 27 and 29 kDa, respectively, aligning with their anticipated theoretical molecular weights. However, CPP-tBLIP-II displayed a lower molecular weight than the expected 29 kDa. Upon cultivating one liter of *E*. *coli* cells, the protein yields were quantified at 9.63 mg, 10.26 mg, and 2.19 mg for tBLIP-II, tBLIP-II-CPP, and CPP-tBLIP-II, respectively.

### Measurement of β-lactamase inhibitory activities of tBLIP-II, tBLIP-II-CPP, and CPP-tBLIP-II

**Fig 4:**
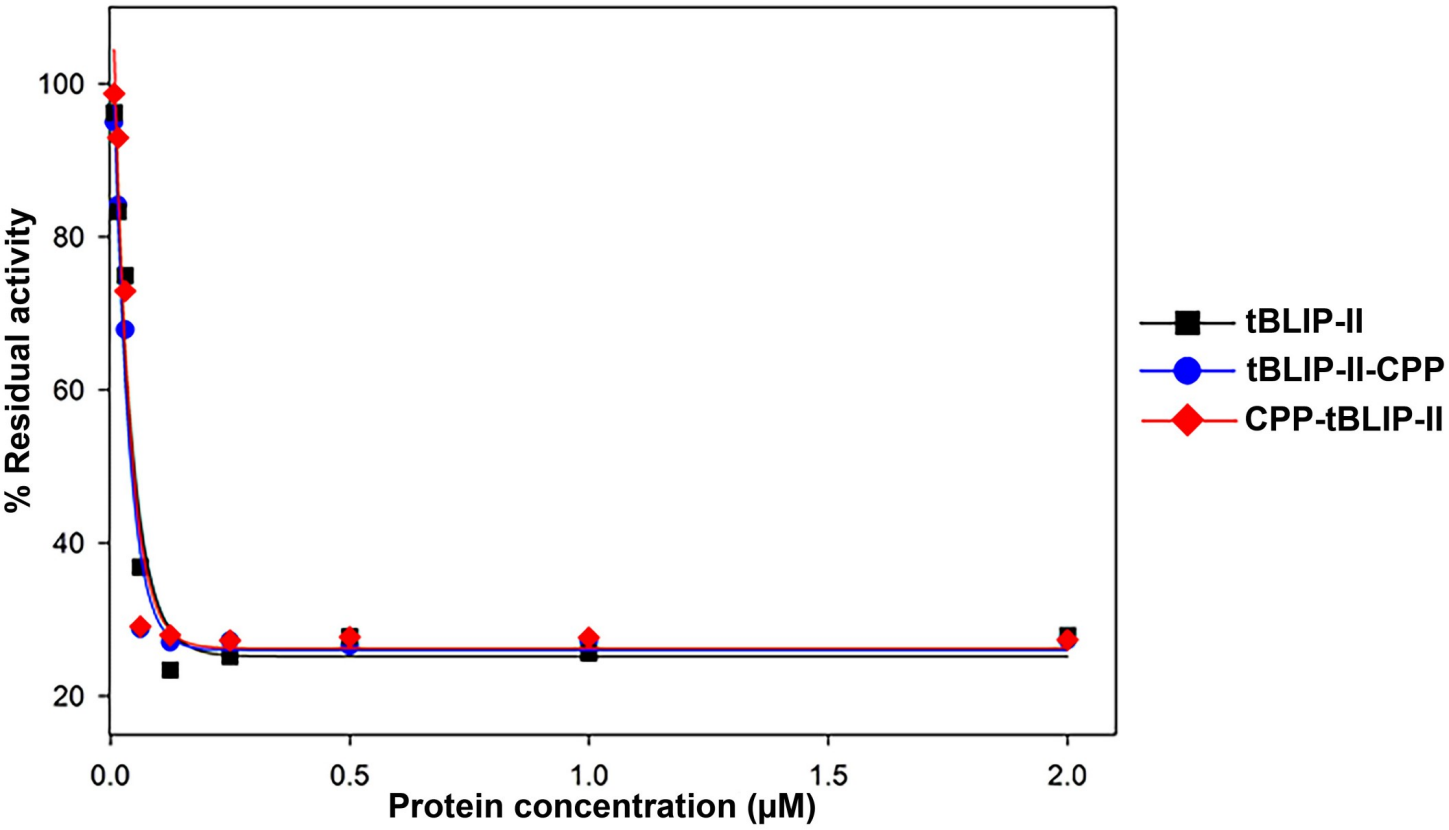
Determination of IC_50_ values for tBLIP-II, tBLIP-II-CPP, and CPP-tBLIP-II. The residual activity of β-lactamase after 15 min pre-incubation with varying concentrations of different inhibitors as monitored by the hydrolysis of nitrocefin.

The β-lactamase inhibitory activities of tBLIP-II, tBLIP-II-CPP, and CPP-tBLIP-II were explored by measuring their ability to inhibit the hydrolysis of nitrocefin by β-lactamase obtained from *B*. *cereus*. All three proteins exhibited similar inhibitory effects on β-lactamase activity. At a concentration of 0.0625 μM for each protein, there was an approximate 70% reduction in β-lactamase activity, and this inhibitory effect remained constant even with increased protein concentration (Fig 4). To quantify the inhibitory activities, the IC_50_ value was determined, which represents the concentration of the inhibitor required to inhibit 50% of the β-lactamase’s specific activity. The IC_50_ values for tBLIP-II, tBLIP-II-CPP, and CPP-tBLIP-II were determined and are shown in Table 3. Both tBLIP-II-CPP and CPP-tBLIP-II exhibited significant inhibitory activity at a similar level as tBLIP-II. However, considering the differences in molecular weight, low expression yield, and comparable inhibitory activity observed in CPP-tBLIP-II, only the tBLIP-II and tBLIP-II-CPP constructs were deemed suitable for further experimental investigations.

**Table 3:** IC_50_ values of tBLIP-II, tBLIP-II-CPP, and CPP-tBLIP-II recombinant proteins.

Recombinant protein	IC_50_ (μM)
tBLIP-II	0.0511 ± 0.0169
tBLIP-II-CPP	0.0435 ± 0.0033
CPP-tBLIP-II	0.0452 ± 0.0063

### Effect of tBLIP-II and tBLIP-II-CPP to increase *K*. *pneumoniae* carrying *bla*_*KPC-2*_ sensitivity to meropenem

**Fig 5:**
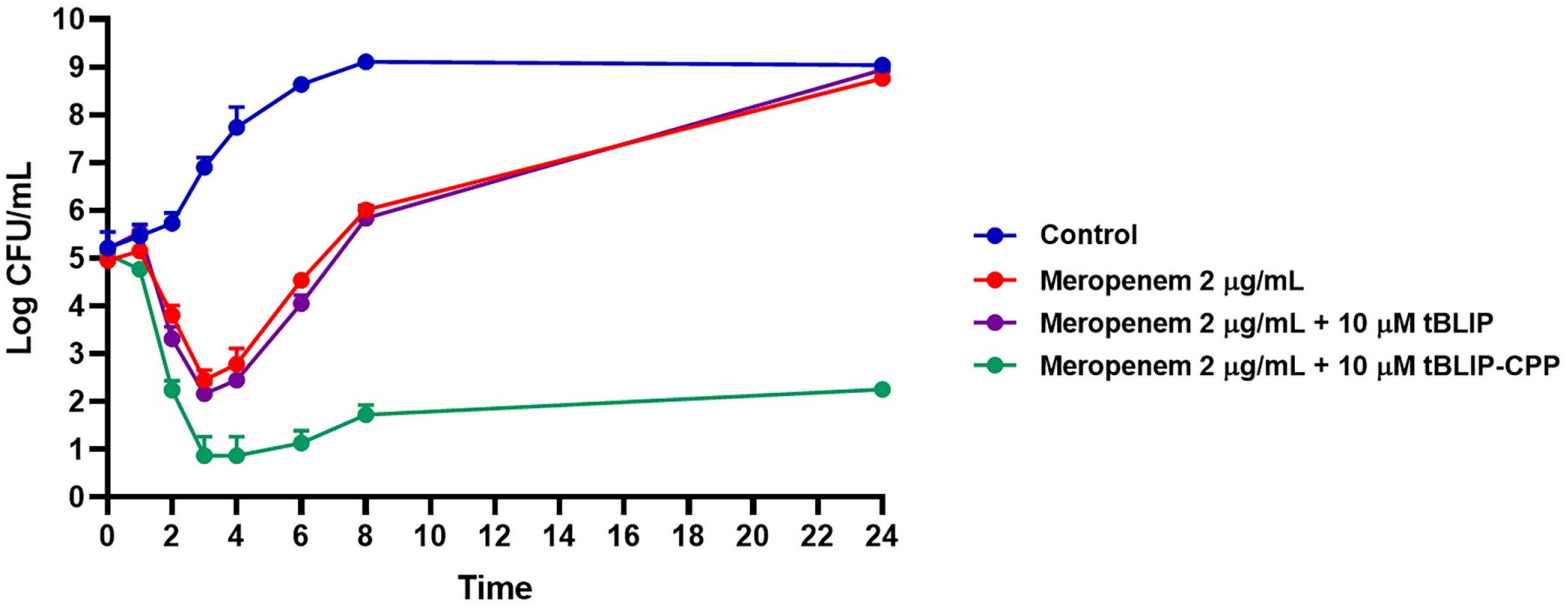
The time-kill curve analysis of combined treatment. KPC-2-producing *K*. *pneumoniae* was treated with the combination of meropenem and recombinant proteins, tBLIP-II and tBLIP-II-CPP, compared to single treatment with meropenem at 0, 1, 2, 3, 4, 6 8, and 24 h.

Based on the inhibitory effect of tBLIP-II against class A carbapenemase, *K*. *pneumoniae* ATCC BAA-1705, a strain known for its KPC-2 positivity, was selected as a model organism to investigate the restoration of meropenem susceptibility. *K*. *pneumoniae* ATCC BAA-2472 was used as the negative control. The MIC assay was performed by combining the various concentrations of meropenem with the recombinant protein at fixed concentrations (10 μM and 20 μM). Notably, the assays involving tBLIP-II and tBLIP-II-CPP alone did not exhibit any antibacterial activity at any concentration (Table in S4 File). Nevertheless, tBLIP-II-CPP at both concentrations showed a substantial 8-fold reduction in the MIC of meropenem, lowering it from 16 to 2 μg/mL against *K*. *pneumoniae* harboring *bla*_KPC-2_, as presented in Table 4. Meanwhile, tBLIP-II at similar concentrations achieved a 2-fold reduction in the meropenem MIC, from 16 to 8 μg/mL. Conversely, none of the proteins were able to reduce the MIC of meropenem against *K*. *pneumoniae* harboring *bla*_NDM-1_, thereby validating the specificity of tBLIP-II towards the KPC-2 enzyme. To further confirm the enhancing effect of the recombinant protein on meropenem, a time-kill curve analysis was performed using *K*. *pneumoniae* ATCC BAA-1705. The results demonstrated that during the initial incubation period, the combination of meropenem with tBLIP-II-CPP exhibited a slightly lower bacterial cell count compared to meropenem treatment alone and combined with the tBLIP-II (Fig 5 and S5 File). Furthermore, the combination of meropenem with tBLIP-II-CPP resulted in more rapid killing throughout 24 h. Noticeably, at 24 h of incubation, the combination of tBLIP-II-CPP and meropenem showed a remarkable decrease in viable cells >2 log10 (CFU/mL)-fold compared to meropenem alone and in combination with tBLIP-II, which exhibited bacterial re-growth. Moreover, the combination treatment of tBLIP-II-CPP and meropenem was effectively bactericidal, reducing the number of viable bacterial cells by >3 log10 CFU/mL over a 24-hour period, relative to the initial inoculum. These findings indicate that tBLIP-II-CPP acts as a potent synergistic adjuvant, effectively restoring the susceptibility of meropenem treatment in *K*. *pneumoniae* carrying *bla*_KPC-2_.

**Table 4:** MICs of meropenem alone or in combination with BLIP-II or BLIP-II-CPP against KPC-2 and NDM-1 producing *K*. *pneumoniae*.

Concentration of protein (μM)		MIC of meropenem (μg/mL)	
		*K*. *pneumoniae*	*K*. *pneumoniae*
		ATCC BAA-1705	ATCC BAA-2472
		(*bla*_KPC-2_ positive)	(*bla*_NDM-1_ positive)
tBLIP-II	0	16	128
	10	8	128
	20	8	128
tBLIP-II-CPP	0	16	128
	10	2	128
	20	2	128

### *In vitro* cytotoxicity evaluation of tBLIP-II and tBLIP-II-CPP against MRC-5 cell line and sRBC

**Fig 6:**
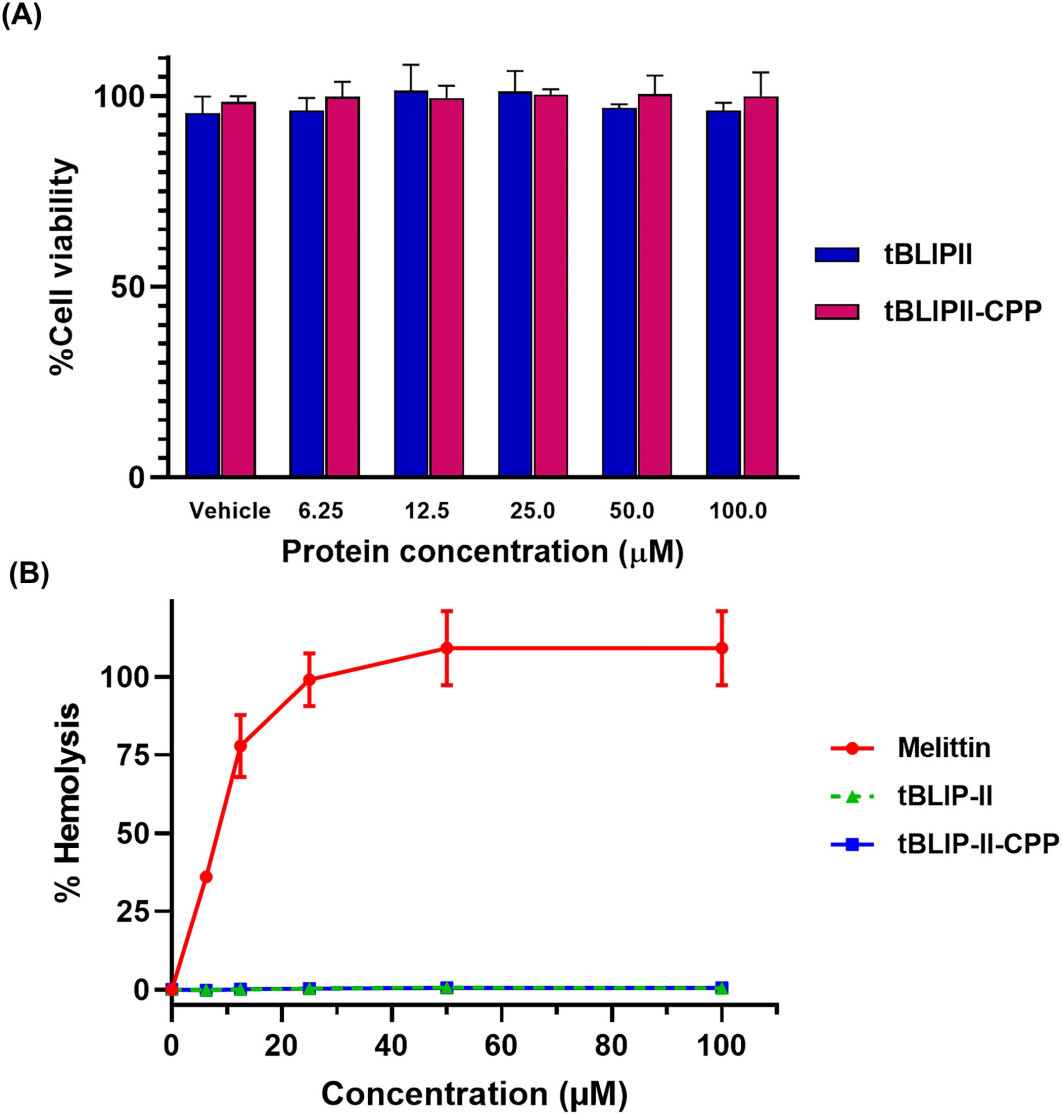
Cytotoxicity and hemolytic activity of the tBLIP-II and tBLIP-II-CPP. (A) The cytotoxic effect of recombinant proteins on human lung fibroblast cell line MRC-5. (B) The hemolysis induction of tBLIP-II and tBLIP-II-CPP on sRBCs. Melittin peptide was used as a positive reference.

To determine the potential cytotoxicity of tBLIP-II and tBLIP-II-CPP towards mammalian cells, two different cell types were tested: the MRC-5 cell line and sRBC. The assessment of *in vitro* cytotoxicity on MRC-5 cells indicated that there was no significant difference in cell viability observed after 24 h of exposure to either tBLIP-II or tBLIP-II-CPP proteins (Fig 6A and S6 File). Furthermore, our investigation of the *in vitro* hemolytic activity of tBLIP-II and tBLIP-II-CPP on sRBC demonstrated minimal hemolytic effects even at a concentration of 100 μM, which was 10 times higher than the concentration required to resensitize meropenem antimicrobial activity (Fig 6B and S6 File). In contrast, melittin, a well-known antimicrobial peptide with high hemolytic activity, exhibited a 50% hemolysis (HL_50_) at a concentration of 8 μM. These findings from both experimental models strongly suggested that tBLIP-II and tBLIP-II-CPP exhibited negligible cytotoxicity in mammalian cells.

## Discussion

The escalating prevalence of carbapenem-resistant Enterobacterales has significantly limited the therapeutic options available for treating infections, necessitating the development of novel resensitizing agents or adjuvants. Within the BLIP protein family, comprising BLIP, BLIP-I, and BLIP-II, BLIP-II has exhibited the highest β-lactamase inhibitory activity against the class A β-lactamases [24, 39]. Characterized as a secreted protein, BLIP-II adopts a seven-bladed β-propeller fold, consisting of three strands and one helix per blade [26]. Extensive investigations have been conducted on the interactions between BLIP-II and two class A β-lactamases, TEM-1 and Bla-1. The co-crystal structure of BLIP-II with TEM-1 has revealed that β-propellers employ their β-turns and/or loops to engage in protein-protein interactions, similar to BLIP. The crucial amino acid residues involved in the interaction with both β-lactamases are nearly identical and encompass Gly49, Asn50, Asn51, Trp53, Tyr73, Asn112, Asp131, Trp152, Tyr19, Asp206, Tyr208, Phe209, Phe230, Glu268, Trp269, and Asn304. Solely two amino acids (Thr57 and Phe74) and four amino acids (Leu91, Asp167, Asp170, and Ile229) exhibit unique interactions with TEM-1 and Bla-1, respectively [51]. Importantly, this study sought to enhance the intracellular permeability of BLIP-II for effective targeting by modifying the tBLIP-II molecules through the fusion of a CPP at either the C-terminus or N-terminus of tBLIP-II. To prevent extracellular secretion, the signal amino acid sequence of the chimeric protein was removed during expression design [39]. Homology modeling results have confirmed that the presence of CPP or a His-tag did not interfere with the amino acid residues involved in β-lactamase enzyme interactions. The expressed proteins, tBLIP-II, tBLIP-II-CPP, and CPP-tBLIP-II, were purified to homogeneity, with yields of 9.63, 10.26, and 2.19 mg/L of culture, respectively. These yields were comparable to previous studies that expressed BLIP proteins in the *E*. *coli* expression system, which ranged from 0.25 to 4.2 mg/L of culture [52]. Notably, the yield of CPP-tBLIP-II was relatively lower than that of the other constructs, potentially attributable to the CPP tag at the N-terminus. Although small peptide tags generally exhibit minimal interference when fused to proteins, they may occasionally exert an adverse influence on the tertiary structure and cause low yield of the chimeric protein [53, 54]. Interestingly, the β-lactamase inhibitory activity of both tBLIP-II-CPP and CPP-tBLIP-II was found to be comparable to that of tBLIP-II as considered from their IC_50_ values, indicating that the fusion of CPP did not impede the active site of tBLIP-II, aligning with the results obtained from homology modeling. These results suggested that the fusion of CPP did not disturb the biological activity of tBLIP-II.

Carbapenemase (KPC) enzymes hydrolyze various β-lactam antibiotics, including commonly used carbapenems for the treatment of severe infections. Unfortunately, KPC enzymes can also develop resistance to commercially available β-lactamase inhibitors which limited the efficacy of these inhibitors. The prevalence and epidemiological status of KPC carbapenemases exhibit significant variation worldwide. *K pneumoniae* is the most common bacterial species harboring KPCs. Noteworthy, patients infected with KPC-producing *K*. *pneumoniae* have a high mortality that exceeds 40% [55, 56]. Regrettably, there is currently no optimal treatment available for infections caused by KPC-producing bacteria. Although combinations of antibiotics are typically employed, they must be tailored to match the remaining susceptibility of the infecting strains [13]. Significantly, KPC-2 is identified as the most frequent carbapenemase in several surveillances [57–59]. The introduction of new β-lactamase inhibitor combinations active against KPC-2 enzymes may offer more comprehensive treatment options. In the present study, the inhibitory β-lactamase activity of the purified tBLIP-II and tBLIP-II-CPP proteins was tested against *K*. *pneumoniae* ATCC BAA-1705 which carries *bla*_KPC-2_. Combined treatment with 10 μM of tBLIP-II-CPP resulted in a decrease in the MIC of meropenem from 16 to 2 μg/mL (8 folds), whereas combining with tBLIP-II at the same concentration decreased the MIC of meropenem from 16 to 8 μg/mL (2 folds). These results demonstrated the superior activity of tBLIP-II-CPP compared with tBLIP-II against KPC-2-producing strain that could lower MIC of meropenem than the CLSI-resistant breakpoint (≥4 μg/mL) [60]. Additionally, these findings implied that CPP could enhance cellular entry across the membrane of tBLIP-II to intracellular targets. A previous study has revealed that co-administration of the expressed BLIP with ampicillin effectively inhibited the growth of β-lactamase-producing *Bacillus subtilis* [48]. Intriguingly, our study showed that treatment of the tBLIP-II alone exhibited no antimicrobial activity, in contrast to a former study in which the BLIP-pVec chimeric protein displayed antimicrobial activity against β-lactamase-producing *E*. *coli*. This discrepancy in antimicrobial effect may be ascribed to the peptide sequence in BLIP-pVec, which acts as an antimicrobial peptide capable of disrupting cell wall/membrane integrity [38]. Furthermore, combined treatment of meropenem with tBLIP-II or tBLIP-II-CPP exhibited no activity against *K*. *pneumoniae* carrying *bla*_NDM-1_, indicating the selective inhibitory action of tBLIP-II against KPC-2, a class A serine β-lactamase, consistent with the observations with BLIPs in the previous studies [27, 61]. Of note, NDM-1 is a class B carbapenemase, which is zinc-dependent, and there are currently no clinically available inhibitors for it [62, 63].

The development of recombinant proteins for clinical applications encounters several challenges, including potential toxicity to mammalian cells. Some peptide sequences possess the capability to hamper the growth of mammalian cells or induce apoptosis [64, 65]. Herein, the cytotoxic effect of tBLIP-II and tBLIP-II-CPP on MRC-5 human lung fibroblasts and sRBCs was investigated. Both proteins exhibited minimal toxicity towards the MRC-5 cell line and displayed very low hemolytic activity rendering them, particularly tBLIP-CPP, promising candidates for clinical applications.

In conclusion, the tBLIP-II-CPP chimeric protein was successfully generated while retaining comparable inhibitory activity to tBLIP-II as described by IC_50_ values. Combined treatment of meropenem with this fusion protein against *K*. *pneumoniae* carrying *bla*_KPC-2_ resulted in a significant reduction of meropenem MIC, thereby enhancing its susceptibility to meropenem. Notably, this combination was more effective when compared with the combination of tBLIP-II and meropenem. As a result, the combination of tBLIP-II-CPP and meropenem could be potentially used in clinical to address the challenge of KPC-2-positive pathogens. Furthermore, tBLIP-II-CPP exhibited negligible hemolytic activity and cytotoxicity, underscoring their potential as potent therapeutic options for addressing drug-resistant bacterial infections. However, further *in vivo* experiments are imperative to validate the efficacy of these combination regimens in the treatment of infections caused by class A carbapenemase-producing bacteria.

## Supporting information

S1 File Predicted model of tBLIP-II generated using I-TASSER. (PDF)

S2 File Predicted model of tBLIP-II-CPP generated using I-TASSER. (PDF)

S3 File Predicted model of CPP-tBLIP-II generated using I-TASSER. (PDF)

S4 File The MIC value of tBLIP-II and tBLIP-II-CPP against *K*. *pneumoniae* ATCC BAA-1705 and ATCC BAA-2472. (PDF)

S5 File The minimal data set used for plotting the kinetic growth curve. (XLSX)

S6 File The results of the cytotoxic and hemolytic activities of tBLIP-II and tBLIP-II-CPP. (XLSX)

S1 Raw image Raw SDS-PAGE gel showing the purity of recombinant proteins. (PDF)
