# Supplementary figures and images for: Enhancing the activity of β-lactamase inhibitory protein-II with cell-penetrating peptide against KPC-2-carrying Klebsiella pneumoniae
Source: PLoS One. 2024 Jan 26;19(1):e0296727. doi: 10.1371/journal.pone.0296727 (PMC10817188; doi:10.1371/journal.pone.0296727)

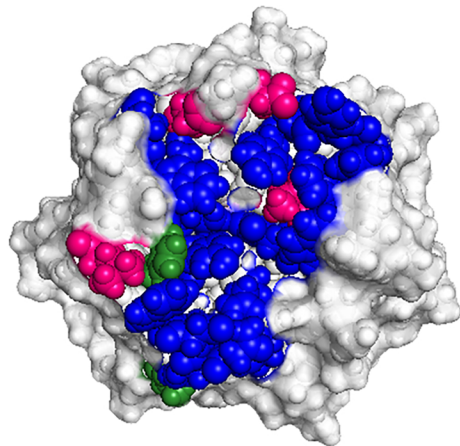

$\phi$   
90°C

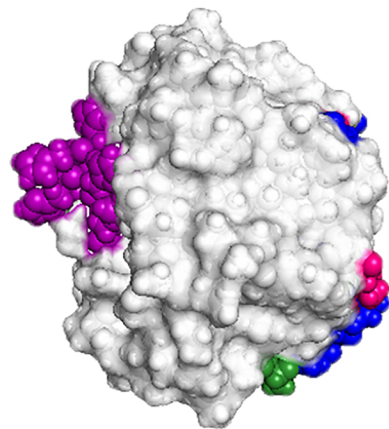

$\phi$   
90°C

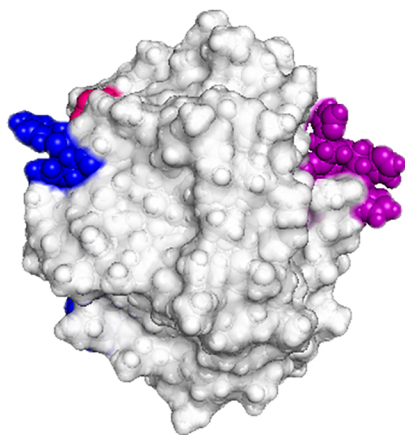

$\phi$   
90°C

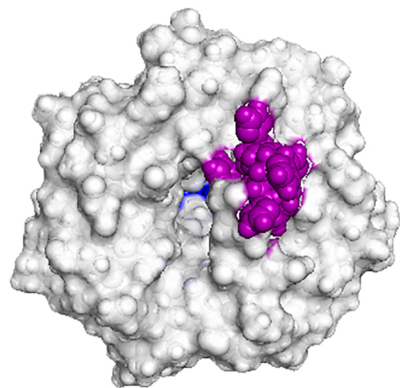

Supplement: S1 File — (PDF) [file pone.0296727.s001.pdf]

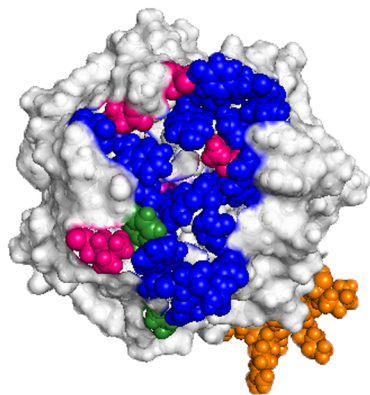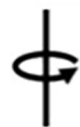

90°C

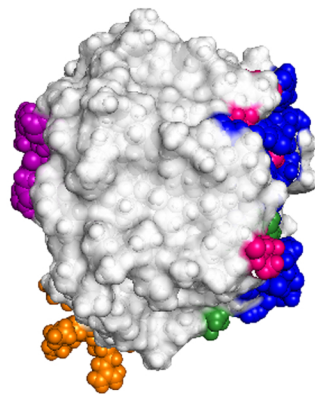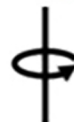

90°C

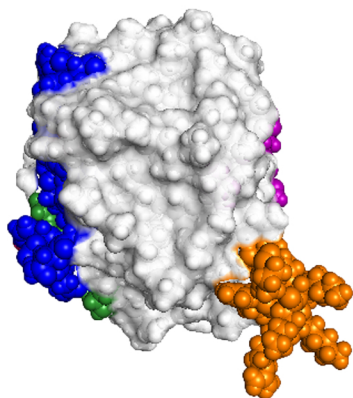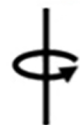

90°C

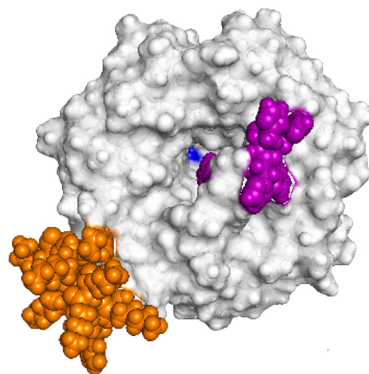

Supplement: S2 File — (PDF) [file pone.0296727.s002.pdf]

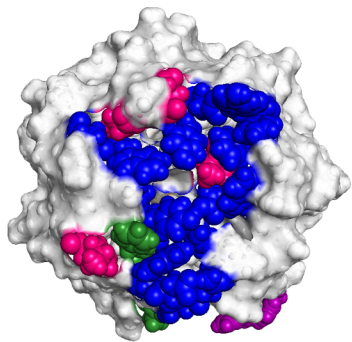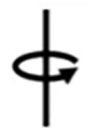

90°C

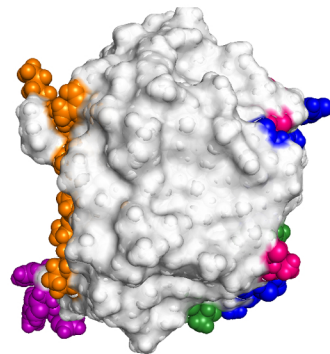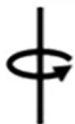

90°C

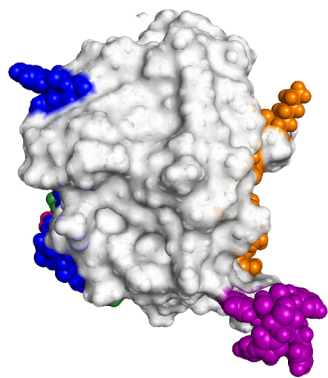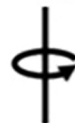

90°C

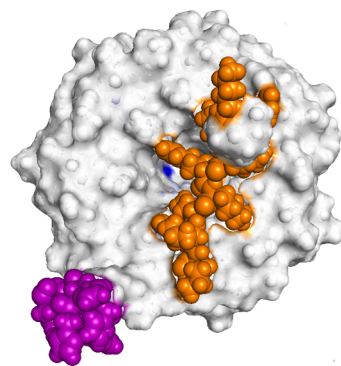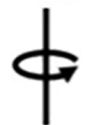

90°C

Supplement: S3 File — (PDF) [file pone.0296727.s003.pdf]
