# Supplementary material for: Enhancing the activity of β-lactamase inhibitory protein-II with cell-penetrating peptide against KPC-2-carrying Klebsiella pneumoniae
Source: PLoS One. 2024 Jan 26;19(1):e0296727. doi: 10.1371/journal.pone.0296727 (PMC10817188; doi:10.1371/journal.pone.0296727)
Supplement: S4 File — (PDF) [file pone.0296727.s004.pdf]

Supplement S4

The MIC value of tBLIP-II and tBLIP-II-CPP against *K. pneumoniae* ATCC BAA-1705 and ATCC BAA-2472

Protein	MIC (µM)	
	K. pneumoniae ATCC BAA-1705	*K. pneumoniae* ATCC BAA-2472
tBLIP-II	>64	>64
tBLIP-II-CPP	>64	>64
