# Supplementary material for: Enhancing the activity of β-lactamase inhibitory protein-II with cell-penetrating peptide against KPC-2-carrying Klebsiella pneumoniae
Source: PLoS One. 2024 Jan 26;19(1):e0296727. doi: 10.1371/journal.pone.0296727 (PMC10817188; doi:10.1371/journal.pone.0296727)
Supplement: S1 Raw image — (PDF) [file pone.0296727.s007.pdf]

Supplement information

S7_Raw image Raw SDS-PAGE gel showing the purity of recombinant proteins.

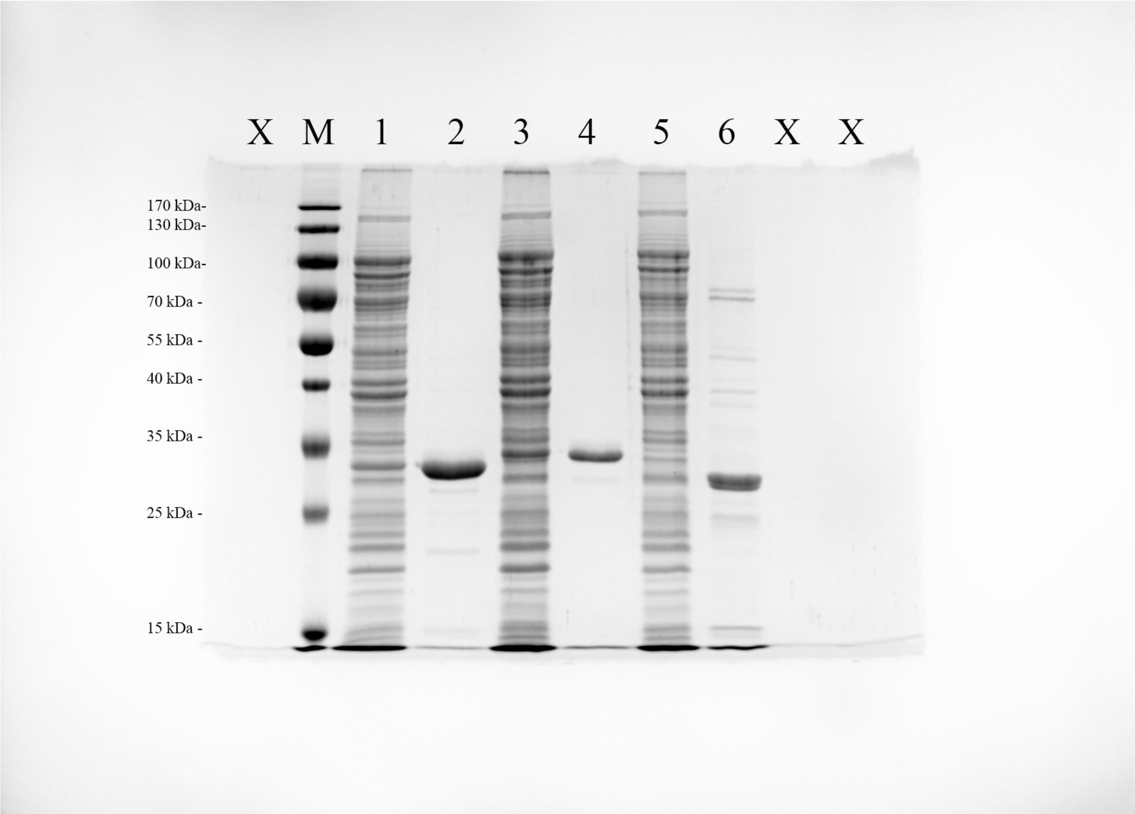

X : Unloaded well
M : Marker
1 : Crude extract of *E. coli* expressing tBLIP-II
2 : Purified tBLIP-II
3 : Crude extract of *E. coli* expressing tBLIP II-CPP
4 : Purified tBLIP-II-CPP
5 : Crude extract of *E. coli* expressing CPP-tBLIP-II
6 : Purified CPP-tBLIP-II
